# Cardiopulmonary testing in adult patients with β-thalassemia major in comparison to healthy subjects

**DOI:** 10.1007/s00277-022-04974-w

**Published:** 2022-09-13

**Authors:** G. Piatti, M. Giuditta, D. Consonni, E. Cassinerio, M. D. Cappellini

**Affiliations:** 1 Department of Pathophysiology and Transplantation, University of Milan and Bronchopneumology Unit, Fondazione IRCCS Ca’ Granda Ospedale Maggiore Policlinico, Via Francesco Sforza 35, Milan, Italy; 2 Department of Clinical Sciences and Community Health, University of Milan and Center for Rare Diseases, Internal Medicine Unit, Fondazione IRCCS Ca’ Granda Ospedale Maggiore Policlinico, Via Francesco Sforza 35, Milan, Italy; 3 Epidemiology Unit, Fondazione IRCCS Ca’ Granda Ospedale Maggiore Policlinico, Via Francesco sforza 35, Milan, Italy

**Keywords:** Cardiopulmonary test, β-Thalassemia, Iron overload, Exercise capacity, Muscular deconditioning

## Abstract

β-Thalassemia patients often have a reduced capacity of exercise and abnormal respiratory function parameters, but the reasons are unclear. In order to identify the causes of the exercise limitation, we performed a cardiopulmonary exercise testing (CPET) in a group of 54 adult β-thalassemia major (TM) patients without pulmonary arterial hypertension and in a group of healthy control subjects. All subjects underwent cardiac echocardiography and carried out pulmonary function tests. TM patients also filled an IPAQ questionnaire on usual physical activity (PA).

Overall, TM patients have a diminished exercise performance in comparison to control subjects. In fact, peak oxygen uptake (V’O_2_ peak), expressing maximum exercise capacity, was decreased in 81.5% of the patients; similarly, anaerobic threshold (V’O_2_@AT) and O_2_ pulse also resulted lowered. In multivariable regression models adjusted for gender, age, BMI, and mean haemoglobin, V’O_2_ peak and O_2_ pulse were positively associated with cardiac iron overload (T2*). No ventilatory limitation to exercise was observed. The most important causes of exercise limitation in these patients were muscular deconditioning and reduced cardiac inotropism due to iron deposition. Only 15/54 (27.8%) TM patients used to perform vigorous physical activity. These results suggest that a program of regular physical activity may be useful to increase the tolerance to effort and therefore to improve the quality of life in these patients.

## Introduction 

Thalassemias are inherited autosomal recessive disorders caused by impaired or absent production of one or more haemoglobin subunits: in β-thalassemia, there is a relative excess of α chains, which can form tetramers and precipitate within the erythroblasts, leading to ineffective erythropoiesis and haemolytic anaemia. In severe forms, symptoms quickly develop after 6 months of age: the subjects develop severe, transfusion-dependent anaemia, hepatosplenomegaly, and skeletal deformities and are prone to infections and fractures [1, 2]. Treatment is usually based on regular transfusions and iron chelation therapy for decreasing iron overload and preventing secondary hemosiderosis effects. Despite the introduction of intensified transfusion regimens and iron chelation therapy with several therapeutic options, exercise intolerance and fatigue still remain common finding in these patients [3, 4].

Most studies [4–6] found a reduced exercise capacity in thalassemia patients and have attributed these findings to a combination of anaemia and iron-mediated cardiotoxicity, but the precise pathophysiological mechanisms have not yet been fully clarified.

The purpose of this study was to assess the causes the relative contribution of the limited exercise capacity in β-thalassemia patients using integrative cardiopulmonary exercise testing (CPET) in a consecutive group of adult β-thalassemia major patients (TM), in which pulmonary arterial hypertension (PAH) was excluded by echocardiographic assessment, in comparison to a healthy control group. In TM patients, the relationship between cardiopulmonary functional capacity and physical activity levels (PA) was also evaluated.

## Patients and methods

We consecutively enrolled 54 adult β-thalassemia major (TM) transfusion-dependent patients regularly followed-up at Center for Rare Diseases of the Policlinico Hospital in Milan, Italy, and 20 healthy sex-, age-, and BMI-matched subjects as a control group.

The TM patients were clinically stable at time of enrolment; none of the patients were smokers, and none had a history, symptoms, or signs of acute or chronic respiratory or cardiac disease. Out of 54, 32 (59.3%) had previously undergone splenectomy. The study was carried out as part of the regular follow-up with patients consent. They were periodically transfused, every 21–25 days, since the first years of life, in order to maintain a pre-transfusional haemoglobin concentration within 9–10 g/dL range. All tests were planned within 7 days after their last transfusions. Within the days of tests, the haemoglobin level was greater than 10 g/dl. Patients were receiving chelation therapy with deferasirox (26 ± 7.6 mg/Kg/day per os; range of dose 10–41 mg/Kg/day) or either deferoxamine (40 mg/Kg/day, 10-12 h sc, 5 days/week) or deferiprone (75 mg/kg/day per os) or both.

All TM patients underwent magnetic resonance (MRI T2*) for iron evaluation in the heart and in the liver within 1 year before the study evaluation. Presence of cardiac iron overload was considered when cardiac T2* value was lower than 20 ms [7]. Normal hepatic T2* value was defined as > 6.3 ms; liver iron concentration (LIC, mg/g dw) was calculated from liver T2* applying the formula [(1/(T2*/1000)] × 0.0254 + 0.202: liver iron overload was estimated for LIC value higher than 4.23 mg/g dw) [7, 8].

Complete M-mode, two dimensional and Doppler echocardiography (GE Vivid 7 and software EchoPAC release 5.2) was performed at rest in TM patients and in the control group to evaluate cardiac function and to exclude pulmonary PAH (PAPs ≥ 35 mmHg). In both groups, spirometric measurements of FVC and FEV_1_ were made in the sitting position with a closed-circuit spirometer (Quark B2, COSMED) as recommended by the American Thoracic Society [9]. Lung diffusion capacity for carbon monoxide (DL_CO_) was measured by an intra-breath analysis (SensorMedics, Yorba Linda CA) corrected for haemoglobin concentration of TM patients [10, 11]. The total lung capacity (TLC) was determined by body plethysmography (Jaeger MS Body, Wurzburg, Germany). A value > 80% of predicted was considered a normal value [12].

TM patients and control subjects underwent an incremental cardiopulmonary exercise test on a cycle ergometer according to the guidelines of the American Thoracic Society [13]. The patients were placed on a cycle ergometer in the upright position: after a 1-min warm-up period at 0 W workload, a ramp protocol of 5–15 W/min was started and continued until achieving peak exercise (reached in approximately 10 min), as assessed using a cut-off > 1.15 for the respiratory exchange ratio (RER). The patients were encouraged to exercise until exhaustion. Oxygen uptake (V’O_2_), carbon dioxide output (V’CO_2_), and minute ventilation (V’E) were measured breath by breath with the online system. Peripheral oxygen saturation (SaO_2_), heart rate (HR), and ECG were monitored continuously; blood pressure was measured every 2 min. The peak V’O_2_ measurements (V’O_2_ peak) were expressed as percent of predicted normal values; O_2_ pulse (O_2_ pulse) was calculated as V’O_2_ divided by HR; anaerobic threshold (V’O_2_@AT) was determined using the V-slope method and expressed as a percent of V’O_2_ max. We used standardized CPET criteria to attribute various patterns of physiological changes during exercise to specific causes [14, 15]: abnormal V’O_2_ peak was considered < 84% of predicted; abnormal cardiac limitation to exercise was defined as an O_2_ pulse ≤ 80% of predicted. Respiratory mechanical limitation to exercise was defined as breathing reserve (BR) < 15% of the maximal voluntary ventilation (MVV), according to the ATS Statement in 2003 [13]. Signs of abnormal gas exchange are a peak exercise HbO_2_ below 88% and/or presence of dyspnoea and V’E/VCO_2_ slope > 34. Muscular deconditioning (MD) was defined as early-onset metabolic acidosis (V’O_2_@AT < 50% of the predicted value), low O_2_ pulse, normal maximum heart rate (HR), and significant BR.

To obtain information about the level of physical activity, a modified version of International Physical Activity Questionnaire (IPAQ) [16] was administered to TM patients. Outcomes measures were average minutes per day of walking, moderate, or vigorous activities; the sum of these variables was computed to obtain a value of total physical activity expressed as metabolic equivalent minutes per week (MET, min/week).

All patients provided written informed consent to the study.

### Statistical analysis

We used chi-squared test (for categorical variables) or Mann–Whitney test (for quantitative variables) to compare TM patients and control subjects. The relationship between cardiac T2* (independent variable, proxy for iron overload) and selected dependent variables was analysed with univariate and multiple linear regression analysis adjusted for gender, age, BMI, and mean Hb. To analyse the relationship of IPAQ and V’O_2_ peak and physical activity (low, moderate, vigorous), we calculated tests for trend using univariate and multivariable linear regression analyses adjusted for the same variables above. Statistical analyses were performed with Stata 17 (StataCorp. 2021).

## Results

In total, 54 consecutive adult transfusion-dependent TM patients and twenty subjects as control group participated in this study. The main characteristics of TM patients are reported in Table 1; the control group consisted of healthy sex-, age-, and BMI matched subjects (10 M, 10F, mean age: 41 ± 6.5 years, BMI 23.8 ± 7 kg/m^2^). Five subjects were excluded from the study, as they were unable to perform correctly spirometry and/or maximal cardiopulmonary exercise testing (RER < 1.15 according to the ATS/ACCP Statement on cardiopulmonary exercise testing, 2003).

**Table 1:** Characteristics of β-thalassemia major patients enrolled (mean values ± standard deviation or numbers)

	Mean ± SD	Range
Gender (males/females)	28/26	-
Age (years)	37.4 ± 6.6	(21–50)
Weight (Kg)	58.9 ± 11	(42–96)
Height (cm)	161.5 ± 8.3	(146–180)
BMI (Kg/m^2^)	22.4 ± 2.8	(17.2–30.2)
Pre-transfusional haemoglobin (g/dL)	9.6 ± 0.5	(8.3–10.5)
Ferritin (ng/mL)	867 ± 879	(146–4367)
Years of transfusion	44.5 ± 7.8	(23–50)
Years of chelation	39 ± 8.5	(20–45)
Cardiac T2* (msec)	37 ± 12	(4–54)
LIC (mg/g dw)	4.3 ± 5	(1–23.3)
Splenectomy (yes/no)	32/22	-

**BMI**, body mass index; **LIC**, liver iron concentration

A total of 46 TM patients were taking a single chelator (deferasirox 33, deferoxamine 12, and deferiprone 1), and 8 patients were taking deferoxamine combined with deferiprone.

None of the TM patients had a symptom history or signs of acute cardiac disease. Echocardiographic measurements in control subjects were within normal values in all cases; no TM patients had relevant systolic or diastolic dysfunction, being ejection fraction (FE) > 55% in all cases, but TM patients showed higher left atrial diameter (LAD), end diastolic diameter of left ventricle (EDDLV), and end systolic diameter of left ventricle (ESDLV) than control subjects, while left ventricular ejection fraction (LVEF) was lower than in the control group (Table 2). Males with TM showed higher end diastolic diameter of left ventricle (EDDLV) and the end systolic diameter of left ventricle (ESDLV) than that of females (both *p* < 0.001). Cardiac iron overload (cardiac T2* ≤ 20 ms.) was observed in 3 of 54 TM patients (5.5%). Hepatic iron overload (LIC > 4.23 mg/g dw) was present in 17 of 54 TM patients (31.5%).

**Table 2:** Echocardiographic findings in β-thalassemia major (TM) patients (*n* = 54) and control subjects (*n* = 20) (mean ± SD)

	TM patients	Controls	*p* value
LAD (mm)	36.5 ± 6.6	20.9 ± 3.1	< 0.001
EDDLV (mL/m^2^)	90.3 ± 18.8	55.7 ± 7.4	< 0.001
ESDLV (mL/m^2^)	32.8 ± 10.8	23.1 ± 5.2	< 0.001
LVEF (%)	63.2 ± 3.5	68.7 ± 2.9	< 0.001

**LAD**, left atrial diameter; **EDDLV**, end diastolic diameter of left ventricle; **ESDLV**, end systolic diameter of left ventricle; **LVEF**, left ventricular ejection fraction

None of the TM patients or the individuals of the control group had symptoms compatible with chronic lung disease. At respiratory function test, 9 TM patients out of 54 showed a restrictive pattern (4 mild and 5 moderate); all control subjects showed respiratory parameters within normal values.

On average, in TM patients, TLC and DL_CO_ were lower than the predicted values (Table 3). TM patients showed lower predicted values in comparison to those of control subjects regarding TLC, FVC, DL_CO_, FEV_1_, and higher values of FEV_1_/FVC. In males with TM, we recorded a percentage of predicted value higher than in females for TLC (*p* = 0.02), FVC (*p* < 0.001), and FEV_1_ (*p* = 0.006).

**Table 3:** Baseline parameters of the respiratory function at rest in beta-thalassemia major (TM) patients (*n* = 54) and control subjects (*n* = 20)(mean ± SD)

	TM patients	Control subjects	*p* value
TLC *(L)*	4.3 ± 1.1	5.1 ± 1.8	0.02
TLC predicted *(%)*	75.5 ± 6.4	92.3 ± 8.5	< 0.001
FEV_1_ *(L)*	2.8 ± 0.3	3.5 ± 0.7	< 0.001
FEV_1_ predicted *(%)*	84 ± 15.5	95 ± 13.2	0.01
FVC *(L)*	3.3 ± 0.9	4.6 ± 0.8	< 0.001
FVC predicted *(%)*	83 ± 4.2	102 ± 9.1	< 0.001
FEV_1_/FVC	0.85 ± 0.1	0.76 ± 0.1	0.001
FEV1/FVC predicted *(%)*	104 ± 15.6	84.5 ± 12.2	< 0.001
DL_CO_ *(mL/min/mmHg)*	18.8 ± 2.1	25.9 ± 7.4	< 0.001
DL_CO_ Predicted *(%)*	64.5 ± 6.3	86.7 ± 7.6	< 0.001

**TLC**, **FEV**_**1**_, **FVC**, and **DLCO** normal values are > 80% of the predicted value, **FEV**_**1**_**/FVC** normal value is > 70% of the predicted value

The CPET variables are presented in Table 4. Based on CPET criteria, the TM patients were limited in exercise capacity due to muscular deconditioning (MD) (*n* = 9), cardiac dysfunction (*n* = 4), mixed cardiovascular dysfunction (CD) and MD (*n* = 17), deficit of lung perfusion (DLP) (*n* = 2), mixed CD and DLP (*n* = 7), and combined DLP and MD (*n* = 5); three patients showed a moderate/severe reduction of exercise capacity due to MD, CD, and DLP. Only 7 out of 54 TM patients showed normal CPET parameters. Four TM patients early stopped the exercise due to fatigue.

**Table 4:** The results of the cardiopulmonary exercise testing (CPET) in β-thalassemia major (TM) patients (*n* = 54) and in control subjects (*n* = 20) (mean ± SD)

	TM patients	Control subjects	*p* value
V’O_2_ peak *(mL/min/kg)*	24.9 ± 5.8	32.6 ± 8.8	< 0.001
V’O_2_ peak predicted *(%)*	70.6 ± 14.9	89.5 ± 6.7	< 0.001
V’O_2_ @AT *(mL/kg/min)*	13.3 ± 3.8	19.1 ± 4.4	< 0.001
V’O_2_ @AT predicted *(%)*	54.7 ± 10.7	65.3 ± 9.8	< 0.001
O_2_ pulse *(L/min)*	9.2 ± 2.2	12.8 ± 3.9	< 0.001
O_2_ pulse predicted *(%)*	81.8 ± 15.6	90.3 ± 10.6	< 0.05
HR max *(bpm)*	159.6 ± 18.5	166.4 ± 15.5	0.13
HR max predicted *(%)*	87.1 ± 9.7	91 ± 5	0.08
V’E peak *(n/min)*	58.7 ± 14.8	52.6 ± 13.7	0.10
V’E/V’CO_2_ slope	30.9 ± 3.6	25 ± 1.2	< 0.001
V’O_2_/Work *(mL/min/watt)*	12.9 ± 1.7	13.2 ± 1.5	0.47
MVV *(L/min)*	102.2 ± 23.2	128.6 ± 18.9	< 0.001
BR *(L/min)*	45.7 ± 17.2	38.9 ± 18.3	0.13
∆ Hb O_2_ sat. *(%)*	1.3 ± 1.3	1.2 ± 1.1	0.75

Normal values are **V’O**_**2**_** peak** > 84%, **V’O**_**2**_** @AT** > 50%, **O**_**2**_** pulse** > 80%, **HR max** > 85% of the predicted values; **V’E peak** < 60 breaths/min.; **V’E/VCO**_**2**_ < 34

Overall, 44 out of 54 TM patients (81.5%) had a reduced exercise capacity as assessed by peak oxygen uptake (V’O_2_ peak) that is below 84% of the predicted value; the anaerobic threshold (V’O_2_ @AT) was reduced (< 50% of the predicted value) in 15/54 (27.8%); O_2_ pulse was < 80% of the predicted value in 28 of 54 patients (51.8%). Differences of gender were observed for V’O_2_ peak (*p* = 0.05), O_2_ pulse (*p* < 0.001), V’E peak (*p* < 0.001), and MVV (*p* = 0.006), with males having greater values. The main parameters obtained from CPET showed lower values for measured V’O_2_ peak, V’O_2_ @AT, O_2_ pulse, MVV, and higher values for V’E/V’CO_2_ slope in TM patients compared with the control group.

No individual of the control group showed limited exercise capacity.

None of the TM patients or subjects of the control group had signs or symptoms of cardiac ischemia during exercise; no ventilatory limitation in exercise was observed; during exercise, no subject of either group had Hb saturation (Hb sat.%) below 88%.

**Fig. 1:**
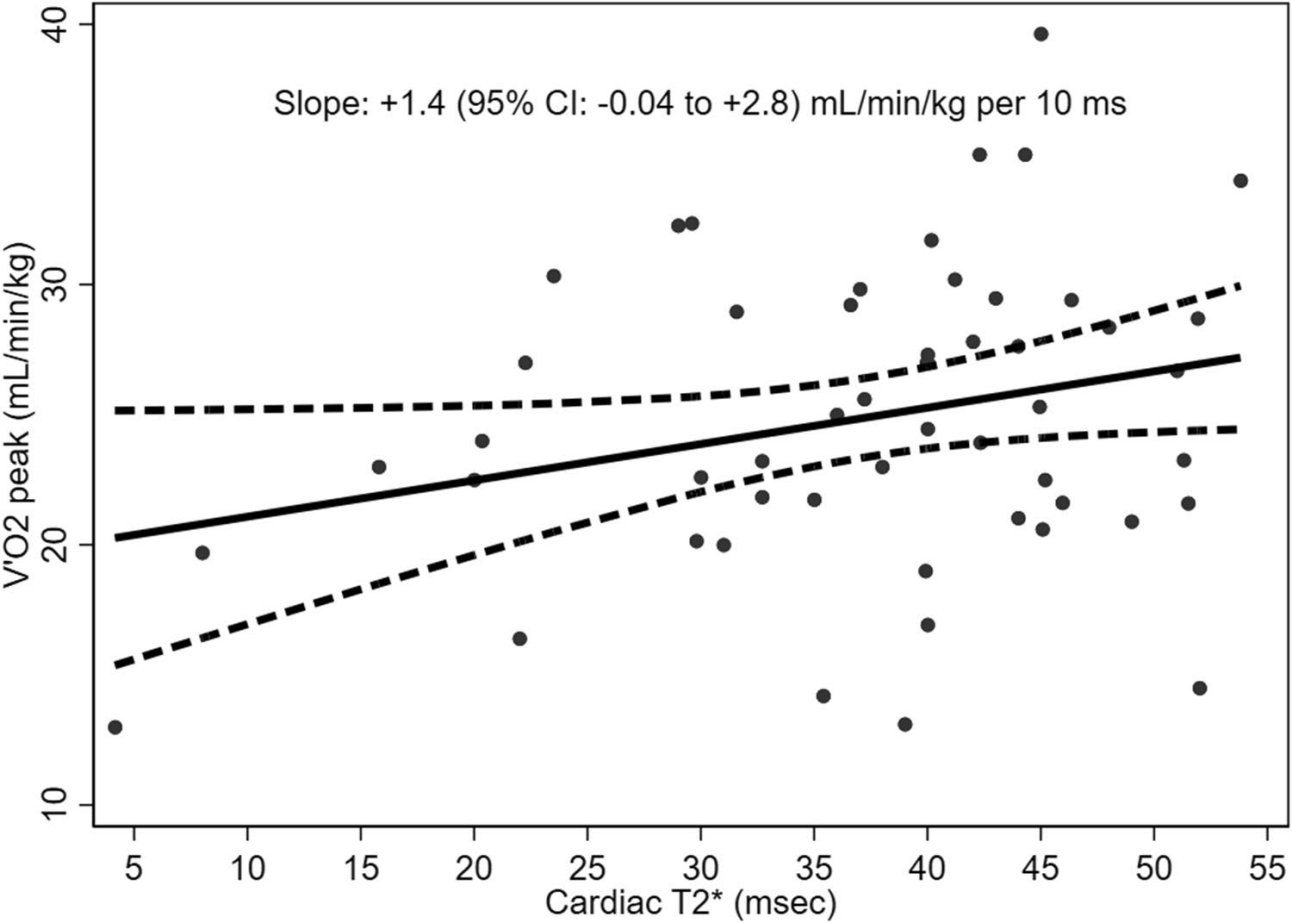
Relationship between V’O_2_ peak and cardiac T2*

**Fig. 2:**
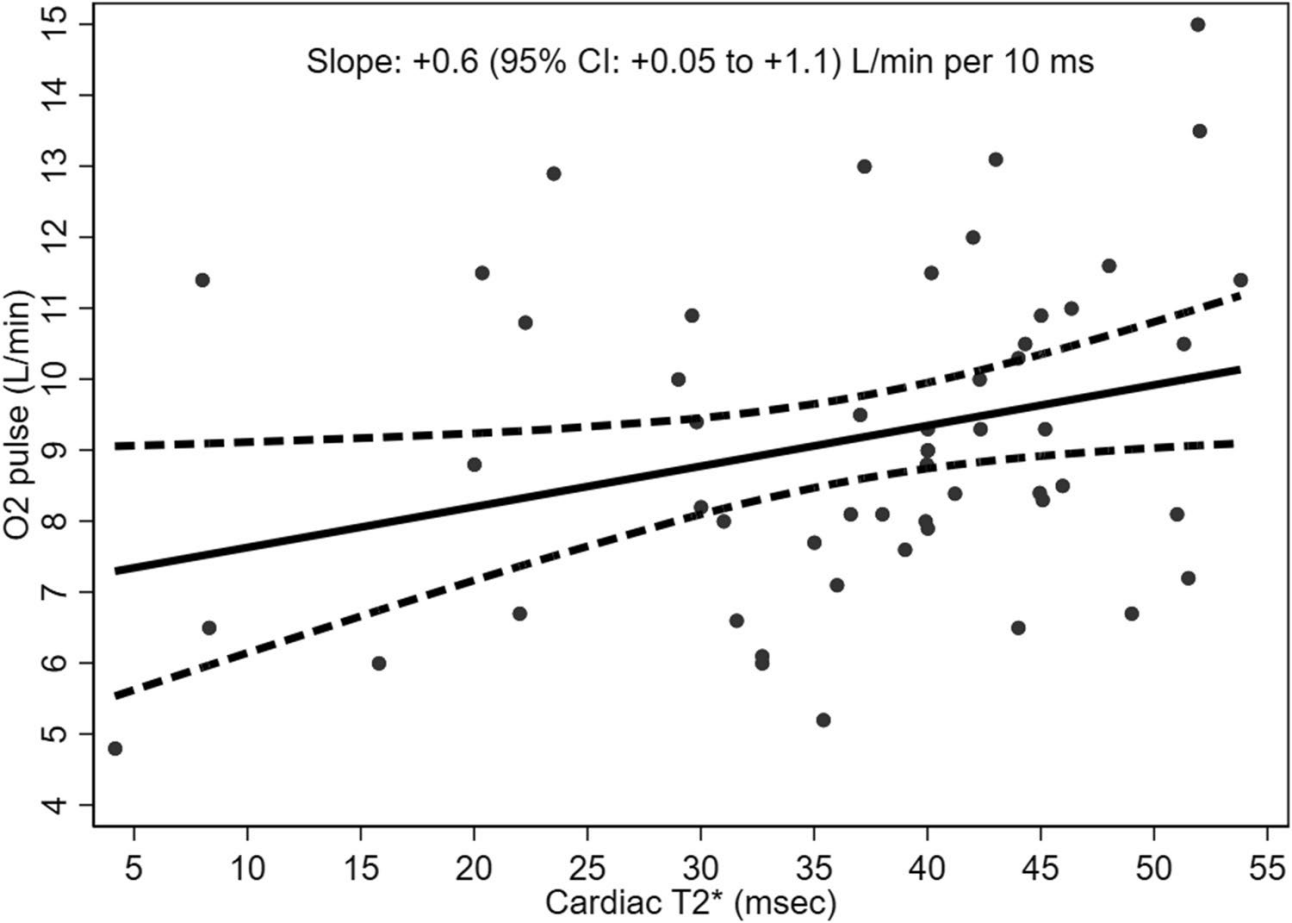
Relationship between O_2_ pulse and cardiac T2*

In TM patients, V’O_2_ peak and O_2_ pulse were positively associated with cardiac T2* (Table 5 and Figs. 1, 2). In multivariable analyses, we confirmed the positive associations between cardiac T2* and V’O_2_ peak and O_2_ pulse, while we found a negative one with V’E peak (Table 5).

**Table 5:** Relationship between cardiac T2* (independent variable, expressed in 10 ms) and selected dependent variables of the cardiopulmonary (CPET) test in β-thalassemia major patients

Dependent variable	Crude slope	95% CI	Adjusted slope*	95% CI*
V’O_2_ peak (mL/min/kg)	+ 1.4	-0.04; + 2.8	+ 1.5	+ 0.1; + 2.9
O_2_ pulse (L/min)	+ 0.6	+ 0.05; + 1.1	+ 0.5	+ 0.09; + 1.0
HRmax (bpm)	+ 1.0	-3.5; + 5.4	+ 1.1	− 3.5; + 5.7
V’E peak (n°/min)	− 0.5	− 7.0; + 5.9	− 6.1	− 11.7; -0.05
MVV (L/min)	+ 0.05	-6.5; + 6.6	+ 0.2	− 1.2; + 3.7

^*^Adjusted for gender, age, BMI, and mean Hb

In a subgroup analysis, TM patients who had undergone splenectomy (18/28 M, 14/26 F) showed some differences compared with those not splenectomised TM patients: in particular, age (40.4 ± 4.3 years vs 33.2 ± 7 years, *p* < 0.001) and left atrial (LAD) diameter (42 ± 2.8 mm vs 35.6 ± 7.5 mm, *p* < 0.001) were higher in splenectomised TM patients while they showed lower ferritin level (607 ± 405 ng/mL vs 1245 ± 1206 ng/mL, *p* = 0.007) and liver iron burden (3.3 ± 4 mg/g dw vs 6 ± 5.8 mg/g dw, *p* = 0.04).

The results of the IPAQ questionnaire showed that TM patients spent little time in total daily PA, without differences between TM patients with normal and reduced lung function. Out of 54 TM patients, only 15 (27.8%) performed a vigorous PA, 31 (57.4%) performed a moderate PA, and 8 (14.8%) low PA. There was a strong association of reported intensity of physical activity with metabolic equivalent minutes per week determined with IPAQ questionnaire and a mild association of reported intensity of physical activity with V’O_2_ peak levels (Table 6): TM patients who reported moderate/high physical activity had very similar V’O_2_ peak (average 25.6 mL/min/kg), which was + 4.5 mL/min/kg higher (*p* = 0.04) than those with low physical activity.

**Table 6:** Relationship between physical activity determined with International Physical Activity Questionnaire (IPAQ) and V’O_2_ peak (mean ± SD) (dependent variables) and intensity of physical activity (independent variable) in β-thalassemia major patients (*n* = 54)

	Intensity of physical activity			
	Low(8)	Moderate(31)	Vigorous (15)	*p* value (test for trend)
Total physical activity (IPAQ) (MET-min/week)	254 ± 89	832 ± 200	1460 ± 283	*P* < *0.001*
V’O_2_ peak measured (mL/min/kg)	21.1 ± 5.3	25.5 ± 4.9	25.7 ± 7.3	*0.12*

## Discussion

This study found a significant impairment in exercise capacity in thalassemia major adult patients, in particular reduced peak oxygen uptake (V’O_2_ peak) in about 80% of subjects. V’O_2_ peak is considered the “gold standard” measure of aerobic fitness and a very strong risk factor inversely associated with morbidity and mortality [17].

Poor physical fitness is a common feature among thalassemia patients. Several reports have shown that patients with thalassemia have lower mean scores of quality of life (QoL) than healthy subjects [18, 19] and that especially the mean scores for physical activity were lower than scores of other dimensions [20].

The two factors that play a greatest contribution to the exercise limitation observed are the abnormal cardiac response and the muscular deconditioning, even if the exercise intolerance in TM patients is likely multifactorial.

Some studies already reported a reduced exercise capacity in TM patients and most attributed these findings to a combination of anaemia and iron-mediated cardiotoxicity [4–6]; other studies mainly attributed the poor physical performance to a limited peripheral muscles response [21, 22].

Indeed, anaemia is a relevant factor in determining the reduction of exercise capacity which is strictly related to blood haemoglobin content, but this factor does not explain entirely this limitation. Anaemia correction by blood transfusions in β-thalassemia patients is associated to a relevant increase of exercise performance, as demonstrated by Benedetto et al. 2015 [23] that found an increase of 82.5 mL/min in V’O_2_ peak for each g/dL of haemoglobin increase.

Nevertheless, there are several other causes of exercise limitation in TM patients, such as the cardiac dysfunction, mainly attributable to cardiac iron deposition and/or a sedentary attitude conditioning a relevant muscle deconditioning or, furthermore, a myopathy of peripheral muscles.

A meta-analysis [24] reveals that cardiac iron overload has a high prevalence in β-thalassemia major and that heart failure and arrhythmias are the most important life-limiting complications in these patients. Myocardial siderosis (cardiac T2* < 20 ms) appears highly related to the risk of heart failure or arrhythmias, thus supporting the validity of myocardial T2* as an early predictor of heart complications [25, 26]. Our results showed that V’O_2_ peak, O_2_ pulse and cardiac LVEF are strongly related to cardiac iron burden T2*, although no relevant echocardiographic abnormalities were found in our group of patients.

Therefore, in addition to the iron-mediated cardiotoxicity, on the basis of CPET criteria to attribute various pattern of physiological changes during exercise to specific causes, we found that the muscular deconditioning is one of the most prevalent causes of a limited exercise capacity in TM patients.

It is known that thalassemia patients are less physically active and have significantly reduced muscle mass compared to the general population [19]. In our study, the filling of IPAQ questionnaire confirms that only a small percentage of them (27.8%) spend time in vigorous intensity activities and that moderate/vigorous PA is associated with best V’O_2_ peak values.

Previously, Nanas et al. [21] assessing respiratory muscle strength performance by maximum static inspiratory pressure (Pimax) found a markedly reduced exercise capacity in a group of TM patients without cardiac disease, in comparison to healthy subjects.

Shapira et al. [27], performing muscle biopsies in TM, confirmed the presence of nonspecific myopathic abnormalities, including moderate variation in fibre size with fibre atrophy and a preponderance of type I muscle fibres.

Stamboulis et al. [28] using nerve and muscle electrophysiology reported a polyneuromyopathy in more than half the TM studied patients.

All these data seem indicate that the limited function of peripheral muscles is one of a major determinants of exercise intolerance in TM patients.

Wagner et al. [29], measuring CPET and vigorous-intensity physical activity using a triaxial accelerometer in 502 subjects from the general population, found a strong positive association with higher V’O_2_ peak and other performance-related CPET parameters, supporting the implementation of higher-intensity aerobic exercise in health promotion. Exercise, especially aerobic activities, demonstrated to improve the QoL in TM patients [30]. Unfortunately, few TM patients perform a regular physical activity due to misconception that anaemia is a limiting factor, and virtually no one is recruited in a program of physical rehabilitation that may help to reduce exercise intolerance. No proper exercise program has been designed yet for these patients, but a planned walking program is recommended to improve their QoL, and a regular aquatic exercise program helps patients to maintain or increase the level of performance [30].

Therefore, some researchers [31] studied the effects of aerobic exercise on iron absorption from the gut and found that regular aerobic exercise reduces it. The use of a regular exercise program combined with drug therapy and blood transfusions can contribute in the treatment of β-thalassemia patients reducing systemic hemosiderosis. The implementation of health education programs, with a special emphasis on physical activity, and providing an exercise program, particularly based on aerobic exercises, along the main treatment of disease, can improve the QoL in TM patients and reduce the associated comorbidities.

The major strength of this study is the large sample of TM patients carefully studied with cardiopulmonary test. The main limitation is the cross-sectional nature of the study. Longitudinal data would be necessary to evaluate the effects of regular physical training program on exercise capacity. In conclusion, these results suggest the opportunity to advise thalassemia patients to engage in regular physical activities to increase the tolerance to effort and therefore to improve the quality of life in these patients.

## Datasets

The datasets generated during the current study are available from the corresponding author on reasonable request.

## References

[1] Taher A, Musallam K, Cappellini MD (2021). Beta-thalassemia. N Engl J Med.

[2] Vji R, Machado RF (2020). Pulmonary complications of hemoglobinopathies. Chest.

[3] Grant GP, Graziano JH, Seaman C (1987). Cardiorespiratory response to exercise in patients with thalassemia major. Am Rev Resp Dis.

[4] Cracowski C, Wuyam B, Klein V, Lévy P (1988). Lung function and exercise capacity in thalassemia major. Eur Resp J.

[5] Sohn EY, Kato R, Noetzli LJ, Gera A (2013). Exercise performance in thalassemia major: correlation with cardiac iron burden. Am J Hematol.

[6] Carpenter JP, Pennell DJ (2009). Cardiopulmonary exercise testing in thalassemia. Int J Cardiovasc Imaging.

[7] Wood JC, Enriquez C, Ghugre N, Tyzka JM, Carson S, Nelson MD, Coates TD (2005). MRI R2 and R2* mapping accurately estimates hepatic iron concentration in transfusion-dependent thalassemia and sickle-cells disease patients. Blood.

[8] Maira D, Cassinerio E, Marcon A, Mancarella M (2017). Progression of liver fibrosis can be controlled by adequate chelation in transfusion-dependent thalassemia (TDT). Ann Hematol.

[9] Quanjer PH, Tammeling GJ, Cotes JE, Of P (1993). Lung volumes and forced expiratory flows. Report of working party standardization of lung function tests, European Community for Steel and Coal. Official Statement of the European Respiratory Society. Eur Resp J.

[10] Cotes J, Dabbs J, Elwood P (1972). Iron-deficiency anaemia: its effects on transfer factor for the lung (diffusing capacity) and ventilation and cardiac frequency during sub-maximal exercise. Clin Sci.

[11] Marrades R, Diaz O, Roca J (1977). Adjustment of DLCO for hemoglobin concentration. Am J Resp Crit Care Med.

[12] Wanger J, Clausen JL, Coates A (2005). Standardization of the measurement of lung volumes. Eur Resp J.

[13] ATS/ACCP Statement on cardiopulmonary exercise testing (2003). AJRCCM.

[14] Milani RV, Lavie CJ, Mehra MR, Ventura HO (2006). Understanding the basics of cardiopulmonary exercise testing. Mayo Clin Proc.

[15] Van Beers EJ, Van Der Plas MN, Nur E (2014). Exercise tolerance, lung function abnormalities, anemia, and cardiothoracic ratio in sickle cell patients. Am J Hematol.

[16] Craig CL, Marshall AL, Sjiostrom M (2003). International physical activity questionnaire: 12 country reliability and validity. Mede Sci Sports Exerc.

[17] Imboden MT, Harber MP, Whaley MH, Finch WH (2018). Cardiorespiratory fitness and mortality in healthy men and women. J Am Coll Cardiol.

[18] Mikelli A, Tsiantis J (2004). Brief report: Depressive symptoms and quality of life in adolescents with beta-thalassemia. J Adolesc.

[19] Gariépy C, Lal A, Fung E (2010). Reduced physical activity in adult and pediatric patients with thalassemia. Blood.

[20] Maheri A, Sadeghi R, Shojaeizadeh D, Tol A (2016). Association between a health-promoting lifestyle and quality of life among adults with beta-thalassemia major. Epid Health.

[21] Nanas S, Vasileiadis I, Dimopoulos S, Sakellariou D (2009). New insight into the exercise intolerance of beta-thalassemia major patients. Scand J Med Sci Sports.

[22] Agostoni P, Cerino M, Palermo P, Magini A (2005). Exercise capacity in patients with beta-thalassemia intermedia. Br J Haematol.

[23] Benedetto D, Rao CM, Cefalù C, Aguglia DO et al. (2015). Effects of blood transfusion on exercise capacity in thalassemia major patients. Plos One May 26: 1–8. 10.1371/journal.pone.0127553 10.1371/journal.pone.0127553 PMC4444349 26010540

[24] Koohi F, Kazemi T, Miri-Moghaddam E (2019). Cardiac complications and iron overload in beta-thalassemia major patients – a systematic review and meta-analysis. Ann Hematol.

[25] Yuksel IO, Koklu E, Kurtoglu E (2016). The association between serum ferritin level, tissue Doppler echocardiography, cardiac T2* MRI, and heart rate recovery in patients with beta-thalassemia major. Acta Cardiol Sin.

[26] Walidiyat PA, Liaw F, Sekarsari D (2017). Evaluation of cardiac and hepatic iron overload in thalassemia major patients with T2* magnetic resonance imaging. Hematology.

[27] Shapira Y, Glick B, Finterbush A, Goldfarb A, Rosenmann E (1990). Myopathological findings in thalassemia major. Eur Neurol.

[28] Stambouilis E, Vlachou N, Drossou-Servou M (2004). Axonal sensorimotor neuropathy in patients with b-thalassemia. J Neurol Neurosurg Psichiatry.

[29] Wagner J, Knaier R, Infanger D, Konigstein K (2021). Novel CPET reference values in healthy adults: association with physical activity. Med Sci Sports Exercise.

[30] Dehkordi AH, Hasani T, Fekri K, Deris F, Etemadifar S (2020). Effects of aquatic exercise on dimensions of quality of life and blood indicators in patients with beta-thalassemia major. Int J Prev Med.

[31] Vashtani SH, Nasem F, Bordar F (2009). The effect of aerobic rehabilitation program on concentration of ferritin, iron, TIBC and cardiovascular operation in the young patients suffering from major thalassemia. J Guilan Univ Med Sci.

